# Consequences of early career nurse burnout: A prospective long-term follow-up on cognitive functions, depressive symptoms, and insomnia

**DOI:** 10.1016/j.eclinm.2020.100565

**Published:** 2020-10-05

**Authors:** Ann Rudman, Lotta Arborelius, Anna Dahlgren, Anna Finnes, Petter Gustavsson

**Affiliations:** Division of Psychology, Department of Clinical Neuroscience, Karolinska Institutet, Nobels väg 9, Solna, Stockholm SE-171 65, Sweden

**Keywords:** Nursing, Professional, Burnout, Consequences, Cohort, longitudinal, Prospective, Early career, Mid-career, Nursing workforce

## Abstract

**Background:**

Burnout is common among nurses and midwives. We examined whether an early career episode of burnout has long-term consequences on; a) cognitive functions, b) symptoms of depression, and/or c) insomnia for nurses a decade after graduation.

**Methods:**

Symptoms of burnout were investigated in an observational longitudinal study of three national cohorts of registered nurses (RNs). Nursing students were recruited from all 26 of Sweden's nursing programs. Burnout was subsequently measured through annual assessment over the first three years post graduation, with one long-term follow-up 11–15 years after graduation. A total of 2474 nurses (62%) consented to participate at follow-up. Burnout was measured using items from the Oldenburg Burnout Inventory, cognitive function by a study specific instrument, depressive symptoms by the Major Depression Inventory, and sleep problems using items from the Karolinska Sleep Questionnaire. We used logistic regression to identify factors associated with consequences of early career burnout, adjusting for concurrent levels at follow up.

**Findings:**

The prevalence of nurses reporting high levels of burnout symptoms at least one of the first three years of working life was 299 (12·3%). High levels of burnout symptoms in early working life were significantly related to more frequent symptoms of cognitive dysfunction, depression, and impaired sleep a decade later when taking current burnout levels into account. After controlling for both current symptoms of burnout and the other outcome variables, nurses with early career burnout still reported more frequent problems with cognitive functions and sleep but not depression.

**Interpretation:**

The results of this study show that the detrimental processes caused by overwhelming or chronic stress start early on in nurses’ careers and thus preventive efforts should preferably be introduced early on (e.g. as part of nursing education and onboarding programs).

**Funding:**

AFA Insurance Grant [number 150284].

**Research in Context**

**Evidence before this study**

We searched Medline, Web of Science, PsycINFO, and CINAHL for studies on long-term follow up of previous burnout among nurses that were published from the inception of each database to March 3, 2020. We found 13 longitudinal studies that have investigated the consequences of burnout in nurses on different outcomes. Twelve studies reported outcomes that were significantly affected by earlier nurse burnout. Most consequences were associated with organizational outcomes such as turnover, absenteeism, and work ability. Only two studies reported on consequences related to nurses’ psychological health but we did not find any study of long-term consequences of burnout on psychological health among nurses. Within the healthcare sector, the supply of nurses is often insufficient and many times financial cuts and demographic changes increase job stress. Thus, high levels of stress and burnout are common among nurses and midwives. The World Health Organization is developing evidence-based guidelines on mental wellbeing in the workplace and the need for rigorous understanding of the development and consequences of these common types of occupational health conditions is pressing. Problems with burnout symptoms for nurses are prevalent already early on in working life. Despite this, no studies have investigated the long-term consequences of elevated levels of early burnout symptoms throughout a professional career.

**Added value of this study**

Early career symptoms of burnout in nurses were shown to have long-term consequences on psychological health with respect to cognitive functions and sleep when controlling for concurrent burnout symptom levels as well as other outcome variables (i.e. cognitive problems, depression, and insomnia). However, nurses with a history of early career burnout did not report more depression than controls at long-term follow-up when controlling for current problems with burnout, cognition and insomnia. The results did not change after controlling for problems with concentration, depressed mood, and sleep quality before professional life, i.e. during nursing education. The current study contributes to new knowledge by showing that job-related stress symptoms have long-term consequences on numerous aspects of psychological functioning. Thus, early career burnout can influence health throughout professional life. Also, by disentangling the overlap between long-term problems with cognitive functions, depression, and insomnia, the results were new and contradict previous literature. An association between early episodes of burnout and depression was expected, however, this relationship was not apparent when controlling for cognition and sleep.

**Implications of all the available evidence**

By identifying when stress-related ill health occurs and the long-term consequences of it, effective timing of interventions can be identified. To avoid negative long-term consequences for nurses’ health, the employer needs to limit stress levels in the workplace and develop methods to ensure sufficient recovery in order to facilitate a sustainable working life. If these factors are not dealt with properly, burnout and its consequences may lead to a further increase in the growing nurse shortage seen worldwide.

Alt-text: Unlabelled box

## 1 Introduction

Symptoms of burnout are common among nurses and midwives [1]. Concurrently, the supply of nurses is insufficient and financial cuts and demographic changes in the healthcare sector increase job stress further [2]. Among these professions, burnout has been associated with high turnover intention, financial loss, and endangered patient safety [3]. These consequences may further increase the growing nurse shortage seen worldwide [4]. Burnout and its negative consequences on nurses and midwives is a societal problem due to their expertise being an essential part of the healthcare sector [5].

In 2019, burnout was included in the International Classification of Diseases (ICD-11) and defined as: *“a syndrome conceptualized as resulting from chronic workplace stress that has not been successfully managed. It is characterized by three dimensions: feelings of energy depletion or exhaustion; increased mental distance from one's job, or feelings of negativism or cynicism related to one's job; and reduced professional efficacy”* [6]. Currently, the World Health Organization (WHO) is developing evidence-based guidelines on how to maintain mental wellbeing in the workplace and the need for rigorous understanding of the development and consequences of these common types of occupational health conditions is pressing.

In Sweden, burnout symptoms were reported by 20% of newly graduated nurses in the first three years of their professional life [7]. Moreover, among those with symptoms of burnout, the prevalence of intention to leave the profession was 27% after one year, 45% after three years, and 43% after five years of employment [8].

Burnout is associated with long recovery periods and elevated ratings of burnout symptoms have been observed up to three years after rehabilitation [9]. Furthermore, even after considerable improvement is seen in burnout symptoms, cognitive problems such as deficits in attention, cognitive inhibition, inhibitory control, and processing speed have been observed in patients with both clinical and non-clinical levels of burnout [10,11]. However, these studies are either cross sectional or based on selected patient populations.

Previous research has consistently shown an association between burnout and depression, with an overlap of symptoms leading to the conclusion that the distinction between the two is not clear [12]. In a review of large-scale longitudinal studies, evidence suggests that depression is a consequence of burnout but not the other way around [13]. There is also evidence that implies that burnout mediates the path from job strain to depression, suggesting burnout as an antecedent to depression [13].

Burnout also shares symptoms with insomnia [14]. In prospective studies, there have been inconsistencies in associations between burnout and sleep [15]. Studies including objective measures of sleep are scarce but one study [16] found that individuals scoring high on burnout symptoms had more short awakenings during sleep and higher levels of impaired sleep efficiency when measured using an electroencephalogram. The long-term consequences of such altered sleep architecture remains to be elucidated through longitudinal studies for a better understanding of the relationship between burnout and insomnia.

When reviewing the literature for consequences of nurse burnout (for details on search see Supplementary Table 1), we found 13 longitudinal studies on different outcomes (see Supplementary Table 2). Twelve studies reported outcomes that were significantly affected by earlier nurse burnout. Most consequences were associated with organizational outcomes such as turnover, absenteeism, and work ability. Only two studies reported on consequences related to nurses’ psychological health, one finding a, in this case, higher risk of depression twelve months after baseline [17] and the other finding an increased risk of post-traumatic stress disorder [18]. Thus, we found no studies investigating the psychological health consequences of burnout among nurses for longer than one year.

The aim of this study was to examine whether an early career episode of burnout in nurses has long-term consequences on; a) cognitive functions, b) symptoms of depression, and/or c) insomnia a decade after graduation. In addition, due to the close link between cognitive difficulties, depression, and insomnia, a further aim was to find out if these outcomes should best be regarded as shared or as unique consequences of early career burnout.

## 2 Methods

### 2.1 Design and participants

**Fig. 1:**
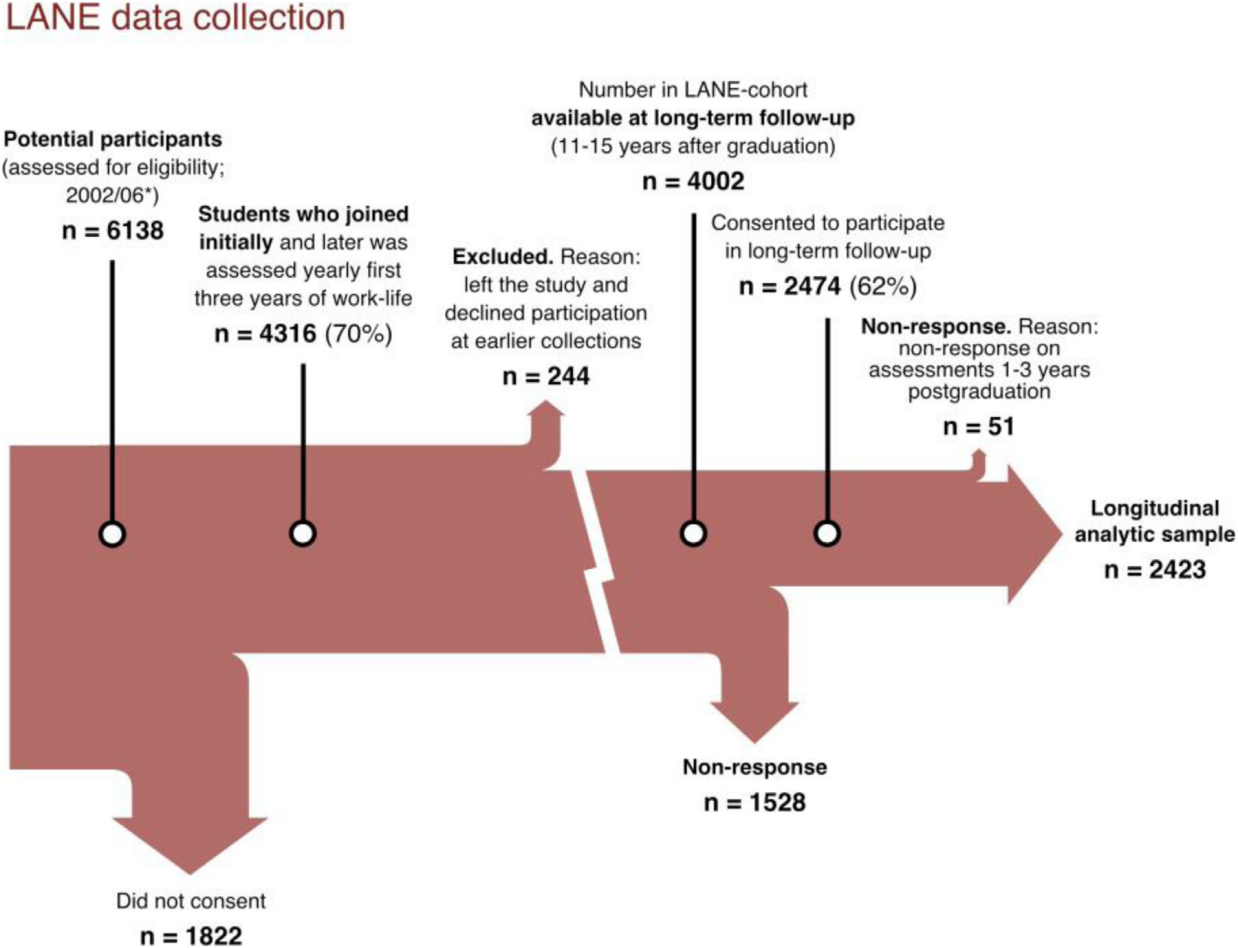
Illustration of the data collections for the three included cohorts, i.e. sample selection and participant recruitment. The “longitudinal” sample consisted of the group of nurses who both responded to the survey during years 1–3 after graduation and to the long-term follow-up 11–15 years after graduation. *All nursing students from all 26 Swedish nursing programs were invited to participate in the study.

In the observational longitudinal study LANE (Longitudinal Analysis of Nursing Education/Entry into work life [19]) symptoms of burnout were investigated in three national cohorts (named by the years of their EXpected graduation – EX2002, EX2004, and EX2006 for students expected to graduate in 2002, 2004, and 2006 respectively). Participants were students recruited from 26 Swedish nursing programs (Fig. 1). Two cohorts were formed in 2002 (one in their sixth semester so graduated in 2002 and the other in their second semester so graduated in 2004) and the third in 2006 (in their sixth semester, just prior to graduation). Burnout was measured in annual assessment waves conducted over the first three working years post graduation with one long-term follow-up eleven to fifteen years after graduation. The study reported in this paper follows a case-control design to estimate associations between exposure (or incidence) of early career burnout and long-term outcomes. As shown in Fig. 1, a total of 2474 nurses (62%) consented to participate in 2017/18, 11–15 years post graduation depending on which year (2002, 2004, or 2006) they graduated from one of Sweden's 26 nursing programs.

When analyzing response rates in the longitudinal data we have previously reported a trend suggesting that males and younger respondents show less participation over time. Initial health status was not found to predict future response rates [19,20]. Dropout analyses were also performed by comparing the 2474 respondents to the 1598 non-respondents in the long-term follow-up regarding cohort, gender, age, general health condition, and sleep quality. The analyses showed that there were more respondents at the long-term follow-up from the EX2002 cohort (70%) than the two other cohorts (60%). Furthermore, more women than men responded to the follow-up. The dropout analysis also showed that the average age was higher among those who responded, i.e. fewer younger participants responded. Poorer health or poorer sleep quality at some point during the previous data collections did not affect the probability of participating in the long-term follow-up. Finally, we have also tested if an early career episode of burnout (ECB) during the first three years of working life predicted participation in the long-term follow-up. We found that those who had experienced an ECB had a higher response rate at follow-up (72%) than those who had not experienced an ECB (64%).

All data were self-reported and collected via postal survey (or in the case of the long-term follow-up via a web survey) with three reminders sent out to non-responders. The reporting of this study is in line with the STROBE recommendations for reporting observational studies [21]. Ethical permission was given by the Regional Research Ethics Committee at Karolinska Institutet, Stockholm, Sweden (Dnr 01–045), and the Regional Ethics Review Board in Stockholm, Sweden (Dnr 04-587 and for the long-term follow-up Dnr 2016 / 793-32).

### 2.2 Predictors, outcomes, and covariables

We focused on burnout symptoms in nurses using yearly assessments for the first three years after graduation as well as at long-term follow-up (Fig. 1, panel with measures [22], [23], [24], [25], [26], [27], [28], [29]). The main variable, job burnout, was measured using items from the Oldenburg Burnout Inventory OLBI [22]. To define ECB we used a one-dimensional sequential-developmental model showing psychometric evidence that different levels of burnout on this scale reflect different phases in the burnout process [23]. This means that “the two main burnout dimensions of exhaustion and dysfunctional coping are ordered sequentially; i.e. initial exhaustion develops into burnout due to dysfunctional coping (cynicism and disengagement)” p. 864 [23]. We assessed psychological health by examining cognitive functions, symptoms of depression, and insomnia at long-term follow-up (Fig. 1, panel). Indicators of previous problems with cognitive functions, symptoms of depression, and sleep quality were measured during the last semester of nursing programs (panel).

### 2.3 Analysis

As cohorts were formed from nursing programs, the data could be regarded as nested within the 26 universities or the 78 classes (3 cohorts * 26 universities). To estimate the magnitude of clustering, intraclass correlations (ICC) were computed for all study variables. All ICCs except one (for academic self-efficacy during education; ICC=1·6%) were under 1%, indicating almost zero influence of clustering (5% is often used as a cut-off). Thus, we have found no indications of problems (or opportunities) seen in nested data and have therefore used logistic regressions on individual data. The prevalence of early career burnout (based on validated cut-off scores [23]) was estimated for each data collection for the total cohort. Those reporting high levels on one or more occasion during the first three years post-graduation were defined as cases and those not reporting high levels of burnout on any of these occasions formed the control group.

Firstly, an estimate of the prevalence for a certain outcome at follow-up was estimated for the total cohort. Secondly, estimates were calculated for the case and control groups. In a third step, an odds ratio was computed in a logistic regression comparing the odds ratio between these two groups on outcome measures at follow-up (OR1). As a fourth step, an additional odds ratio was calculated using logistic regression, now controlling for ongoing episodes of burnout at follow-up (OR2). In a fifth step, new odds ratios (OR3) were calculated for each outcome controlling for the other two (i.e. cognitive functions, symptoms of depression, and sleep problems respectively, as well as current levels of burnout). In a final step, indicators of previous problems (with cognitive functions, symptoms of depression, and sleep problems respectively) measured during the last semester of the nursing education program were added to the logistic regression described in the fifth step, and new odds ratios (OR4) were calculated. Age, sex, and cohort were included as control variables in all logistic regression analyses.

### 2.4 Role of the funding source

The funding source AFA Insurance [Grant number 150284] had no participation in any steps of conducting the study including design of the study, collection or management of data, data analysis and interpretation, writing the manuscript, or deciding to submit it for publication. AR and PG have been involved in all data collections since 2001 and are responsible for and have access to all the full longitudinal data. All authors were involved and responsible for the decision to submit the manuscript.

## 3 Results

In the long-term follow-up sample, 2225 participants (90%) were female and 1409 participants (57%) were specialist-trained nurses. The age range was as follows; 1014 (41%) were aged under 39, 865 (35%) were aged 40–49, and 643 (26%) were aged over 50 years. The majority of participants (73%) had children who live at home and an even higher number were parents. Participants in the long-term follow-up were more likely to be female and of an older age. We calculated the number of participants with missing data for each outcome variable (i.e. cognitive functions, symptoms of depression, and insomnia) at long term follow up. Numbers of missing data were 15 for concentration (0·06%), eight for depression (0·03%), and 14 for insomnia (0·06%).

### 3.1 Burnout

**Fig. 2:**
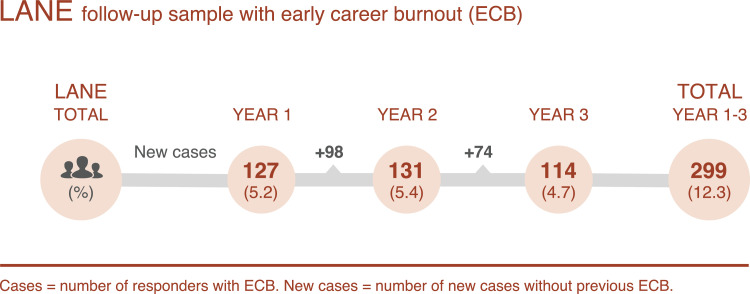
Prevalence of high symptoms of early career burnout (ECB) one, two, and three years after graduation in the longitudinal follow-up sample (*n* = 2423).

High levels of burnout symptoms early in the career, i.e. one, two, or three years after graduation, are shown in Fig. 2. An episode of high symptom levels of burnout is here named Early Career Burnout (ECB), denoting early in relation to the long-term follow-up 11–15 years post graduation. The prevalence of ECB one, two, and three years after graduation was 127 (5·2%), 131 (5·4%), and 114 (4·7%), respectively. The number of nurses in the three cohorts reporting very high symptoms of burnout at least one of the first three years of working life was 299 (12·3%).

### 3.2 Cognitive problems

**Fig. 3:**
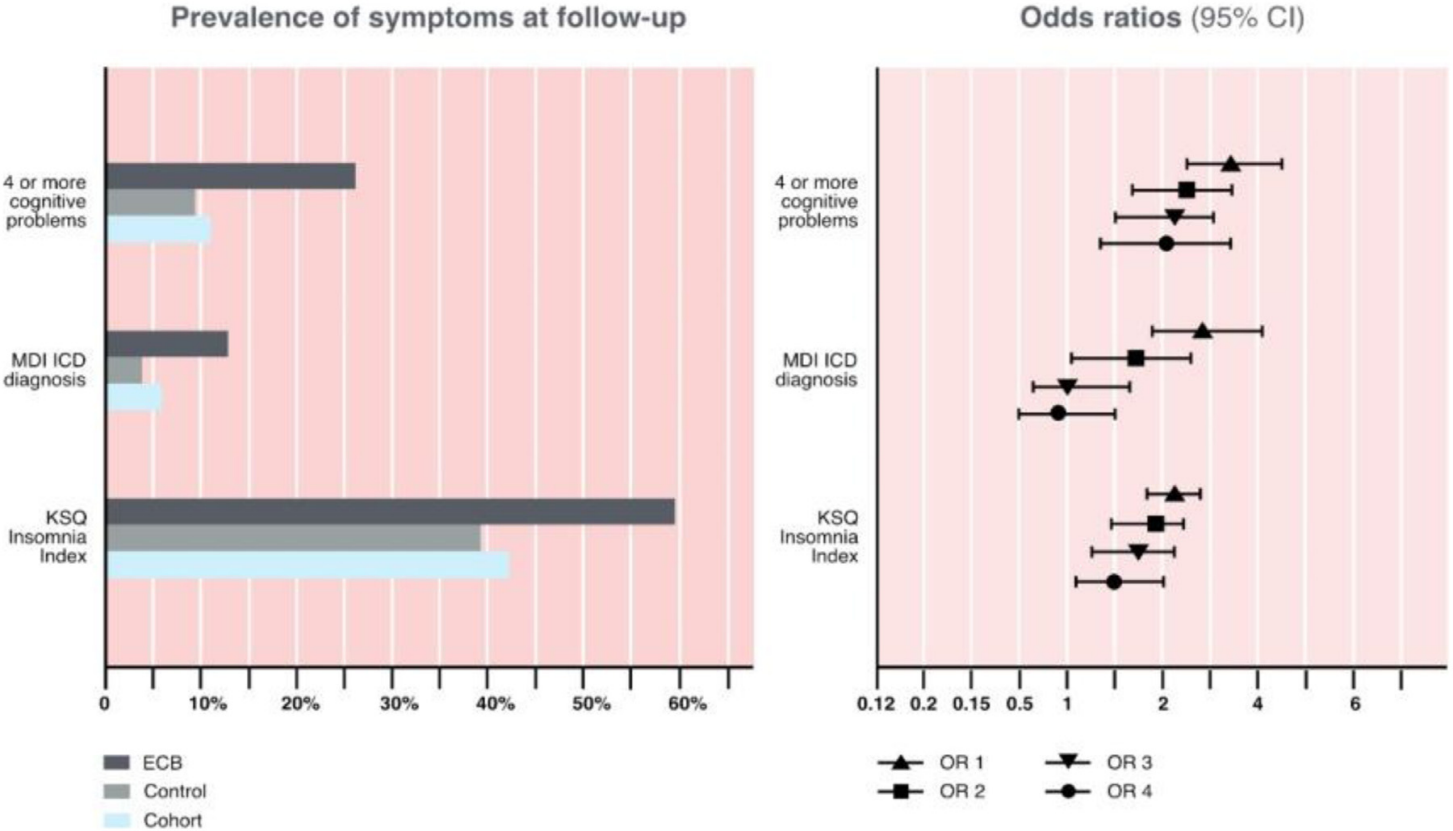
Comparing symptoms among nurses with or without a previous early career episode of burnout at long-term follow-up: prevalence and odds ratios (point and confidence) for cognitive problems (number of cases with four or more cognitive problems, in the last four weeks), depression (MDI ICD diagnosis, in the last two weeks), and sleep problems (KSQ Insomina index). Cohort% = prevalence in the total sample; ECB% = prevalence in the subsample with a history of early career burnout (ECB); control% = prevalence in the subsample without a history of ECB. OR 1 = without correction for concurrent symptoms. OR 2 = corrected for ongoing episode of job burnout. OR 3 = corrected for ongoing episode of job burnout and the other symptoms at 11–14 years post graduation i.e. cognitive problems, depression, and sleep problems respectively. OR 4 = corrected for ongoing episode of job burnout and the other symptoms at 11–14 years post graduation i.e. cognitive problems, depression, and sleep problems respectively and indicators of previous problems (with cognitive functions, symptoms of depression, and sleep problems respectively) measured during the last semester of the nursing education program. Age, sex, and cohort were included as control variables in all logistic regression analyses, thus all odds ratios (OR1-OR4) were controlled for age, sex, and cohort.

At follow-up, 275 (11·4%) of the nurses reported that they often had four or more cognitive problems. Having at least four different cognitive problems was nearly three and a half times more common in nurses who had experienced ECB than in nurses with no history of ECB when controlling for age, gender, and cohort. This effect was also still significant after correcting for ongoing episodes of burnout at follow-up (Fig. 3, Supplementary Table 3).

### 3.3 Symptoms of depression

At follow-up, 138 (5·7%) of nurses fulfilled the criteria for depression. Almost three times as many nurses with a history of ECB got this diagnosis than the controls when controlling for age, gender, and cohort; an effect that was still significant after also correcting for ongoing episodes of burnout at follow-up (Fig. 3, Supplementary Table 3).

### 3.4 Sleep

In the whole cohort, 1020 (42·2%) of the nurses reported symptoms of insomnia at follow-up. Significantly more nurses with an episode of ECB reported insomnia symptoms than the controls, even after controlling for age, gender, cohort, and an ongoing episode of burnout (Fig. 3, Supplementary Table 3).

### 3.5 Disentangling the overlap between cognitive problems, depression, and sleep problems

Taken together, we found that nurses who had experienced an episode of ECB reported significantly more problems with cognitive function, depression, and insomnia at follow-up. As these problems are often linked together, odds ratios were calculated for each symptom controlling for the other two (as well as current levels of burnout). After controlling for age, gender, cohort, and current problems with burnout, depression, and insomnia, nurses with a history of ECB still reported more frequent problems than controls with cognitive functions (OR 3 = 2·185; 95% CI: 1·519–3·142, see Supplementary Table 3). Nurses with a history of ECB also reported more frequent problems than controls with insomnia after controlling for age, gender, cohort, and current problems with burnout, cognitive problems, and depression, (OR 3 = 1·723; 95% CI: 1·315–2·258). However, after controlling for age, gender, cohort, and current symptoms of burnout, cognitive problems, and insomnia, nurses with a history of ECB did not report higher levels of depression than controls at follow-up (OR 3 = 1·038; 95% CI: 0·631–1·706, Supplementary Table 3).

The longitudinal associations between ECB and possible consequences may reflect a stability association between risk factors and outcomes. In order to control for this, indicators of sleep quality, depressive mood, and concentration difficulties taken from data collected during the last semester of the nursing education were entered into the regression equations. Thus, after controlling for age, gender, cohort, problems with concentration before graduation, and current problems with burnout, depression, and sleep, nurses with a history of ECB still reported more frequent problems with cognitive functions at follow-up than controls (OR 4 = 2·155; 95% CI: 1·365–3·402, see Supplementary Table 3). Note that this particular analysis was only possible to do using data from two of the cohorts i.e. EX2002 and EX2004 due to the measure not being included in the EX2006 cohort at education. The results were the same for insomnia. Thus, after controlling for age, gender, cohort, problems with sleep quality before graduation, and current problems with burnout, cognitive problems, and depression, nurses with a history of ECB still reported more frequent problems with insomnia at follow-up than controls (OR 4 = 1·540; 95% CI: 1·170–2·028, Supplementary Table 3). Finally, after controlling for age, gender, cohort, problems with low mood before graduation, and current problems with burnout, cognitive problems and insomnia, nurses with a history of ECB did not report higher rates of depression than controls at follow-up (OR 4 = 0·836; 95% CI: 0·497–1·405, Supplementary Table 3).

## 4 Discussion

The aim of this study was to find out whether ECB symptoms in nurses may have long-term consequences on psychological health with respect to cognitive functions, depression and insomnia. The prevalence of nurses who reported high levels of burnout symptoms during at least one of their first three years of working life was 299 (12·3%). High levels of burnout symptoms in early professional life (compared to no symptoms) was significantly related to more frequent cognitive dysfunctions, symptoms of depression, and insomnia a decade later when taking current burnout levels into account. In addition, after controlling for both current problems with burnout, and the other outcome variables (i.e. cognitive problems, depression, and insomnia respectively), nurses with a history of ECB still reported more frequent problems with cognitive functions and insomnia. However, nurses with ECB did not report more depression compared to controls at long-term follow-up when controlling for current problems with cognition and insomnia. These results also remained the same after controlling for problems with concentration, depressive symptoms, and sleep quality measured during nursing education. The current study adds to the knowledge by showing that job-related stress symptoms had long-term consequences on numerous aspects of psychological functioning. Thus, early career stress can influence health throughout the career. Also, by disentangling the overlap between long-term problems with cognitive functions, depression, and insomnia, the results were new and contradictory to previous literature. For example, an association between ECB episodes and depression at follow-up was expected [13,15]. However, this relationship did not hold up when controlling for concurrent burnout, cognition, and insomnia.

These results can be generalized to occupational burnout also in other settings and contribute to the understanding of the progression of burnout symptoms over time. Although the association between burnout and depression has been discussed and their temporal link questioned [12], previous longitudinal and diagnostic studies have consistently found depression to be a probable consequence of burnout [13,15]. However, our results show that neurocognitive problems and insomnia, but not depressive symptoms, were unique consequences of ECB. Previous longitudinal research finding a temporal link between burnout and depression has been interpreted to point to an association between burnout and the core symptoms of depression (i.e. low mood, sadness, and loss of initiative). However, the results presented here indicate that such an association might not exist. As low mood, sadness, and loss of initiative together with insomnia and neurocognitive problems are part of the depression syndrome, it is not clear if all of these or specific symptoms should be seen as probable consequences of burnout. The broad content of depression-related symptoms captured in these instruments opens up for an alternative interpretation of previously found temporal associations between burnout and depression. Thus, the present study points to sleep and neurocognitive problems as being unique consequences of ECB and that the previously published longitudinal findings might also be interpreted as reflecting these associations. The question is then if the present results uncover an association between burnout and a subtype of depression (dominated by neurocognitive and sleep dysfunctions) or if the results should be interpreted as reflecting unique consequences not associated with depression at all (although symptoms are included in depression inventories). We propose that it might be fruitful to think about neurocognitive and sleep dysfunctions as unique consequences of burnout.

The results are also supported by reports from clinical settings where patients with burnout often complain about long-term cognitive problems even after considerable improvement in burnout symptoms [10,11]. In addition, according to the allostatic load model, i.e. the wear-and-tear process of the brain and body over time [30] by repeated stress responses in combination with failing recovery, severe stress may lead to neuronal atrophy of limbic brain structures such as the hippocampus and the prefrontal cortex [30]. These brain structures support a variety of functions including learning, memory, and other cognitive processes. A lack of recovery after the stressor is over can lead to persisting cognitive impairment and stress-related psychiatric disorders [31]. Thus, changes in brain structures that may occur as a result of an episode of ECB could explain the presence of cognitive symptoms several years after the event.

When controlling for current burnout as well as depression and cognitive symptoms, our results show an increased likelihood of having insomnia at follow-up among those with an episode of ECB. Despite the increased need for recovery in situations with high levels of stress, those same situations are more likely to be associated with impaired recovery (such as impaired detachment from thoughts of work during free-time, impaired sleep, and lack of physical activity), referred to as the “recovery paradox” [32]. The lack of detachment from stressors probably plays a role in this and can sustain stress reactions and lead to a dysregulation of the autonomic nervous system [33]. Due to impaired recovery after a burnout episode possibly playing a central role in the development of any of the outcome measures, future studies should examine factors that could affect employees’ possibilities to recover during time off, such as family situation and work hours. In a previous cross-sectional study, we have shown that shift combinations with quick returns (less than 11 h between shifts) were associated with impaired sleep and recovery [34]. Currently, there is a lack of studies showing the long-term effects of exposure to severe burnout symptoms on sleep, thus our findings contribute towards new knowledge indicating that an early episode may contribute to behavioral and/or physiological changes affecting sleep.

One strength of this study is that the data collection covers three consecutive years after graduation and a long-term follow-up more than a decade later. The study was also based on three independent national cohorts with good response rates in the three first data collections taking place during the early career stage, and these data were compared to the national population of newly graduating nurses and found to be representative [19]. The selection effects in the long-term follow-up were small and since the cohorts were representative at the formation, we conclude that the results are not severely misleading. The sample of nurses is controlled by design, i.e. they received approximately the same education, entered working life at the same time, and are in about the same position in life. The estimates were robust in that they were consistent at item and scale levels and remained the same despite controlling for simultaneous problems. Also, the incidence was measured with repeated measurements over three years and that follow-up was longer than 10 years. One major limitation was the lack of information on what happened in burnout levels and outcomes during the time between follow-up years and the lack of knowledge when the consequences occurred. Another limitation of the study was that all data was self-reported and that the assessment of, for example, cognition can therefore be biased by perceptive insufficiency and/or the subject of socially desirable answers. Similarly, the self-report measures of cognition, depression, and sleep lack other supplementary assessment methods such as neuropsychological memory tests, validated diagnostic gold standard for depression and physiological sleep quality tests (e.g. sleep watches).

The results of this study show that the detrimental processes caused by overwhelming or chronic stress start early on in nurses’ careers and thus preventive efforts should preferably be introduced early on (e.g. as part of nursing education and onboarding programs). By identifying when stress-related ill health occurs and the long-term consequences of it, effective timing of interventions can be identified. To avoid negative long-term consequences for nurses’ health, employers need to limit stress levels in the workplace and develop methods to ensure sufficient recovery from stress-related ill health in order to facilitate a sustainable working life. The consequences of burnout will lead to a further increase in the growing nurse shortage seen worldwide.

## Contributors

AR and PG conceived and designed the study, collected, analyzed the data, and tabulated the results. AR, AF, LA, and PG did the literature search, assessed the articles and synthesized the results. AR, PG, LA, AD, and AF interpreted the results and wrote the first draft of the manuscript. All authors critically revised the manuscript, approved the final version, and meet the criteria for authorship.

**Panel: Instruments**

**Burnout**

The main variable, job burnout, was measured using items from the Oldenburg Burnout Inventory OLBI [22]. The instrument comprises the dimensions exhaustion and disengagement. Exhaustion indicates intensive physical, affective, and cognitive strain, resulting in a tired state, and disengagement indicates the process of distancing oneself from one's work, resulting in negative attitudes towards work in general. Responses were given on a four-point scale: 1 “Does not apply at all”, 2 “Does not apply very well”, 3 “Applies well”, and 4 “Applies completely”. A mean value was computed that ranged from 1 to 4. In this analysis we have used the cut-off that Gustavsson et al. [23] established for severe burnout symptoms (combining scores from seven items of exhaustion and disengagement), i.e. an average value above three on the index. Reliability for the measurement of burnout in terms of internal consistency showed a Cronbach's alpha of 0·79 after one year in the profession, 0·82 after two years in the profession and 0·83 after three years in the profession. The Cronbach's alpha at 11–15 years in the profession was 0·84.

**Cognitive functions**

Eleven items hypothesized to measure respondents’ memory and concentration capacity were constructed (see Supplementary Table 4). The items were inspired by dementia-related items in the Swedish BETULA-study [24]. Respondents were given the options 1 “Every day”, 2 “Several times a week”, 3 “A few times a week”, 4 “Occasionally”, and 5 “Never” during the last four weeks. The cut off for having high levels of symptoms was set at “Every day” and “Several times a week”. The Cronbach's alpha at 11–15 years in the profession was 0·95.

**Depressive symptoms**

Depressive symptoms were measured using the Major Depression Inventory (MDI) [25]. MDI is a measure of depressive symptomatology and can be used to indicate an ICD-10 diagnosis of moderate to severe depression. Based on a person's response on the 10 items with the time frame during the last two weeks, a score (no diagnosis vs. diagnosis) was calculated based on a diagnostic algorithm [25]. The Cronbach's alpha at 11–15 years in the profession was 0·91.

**Insomnia**

Insomnia was measured using items from the Karolinska Sleep Questionnaire (KSQ) [26]. An insomnia index was formed based on the items “difficulties falling asleep”, “difficulties maintaining sleep”, “premature awakening”, and “disturbed sleep”. Respondents were given the options 1 “Almost every day”, 2 “Many times per week”, 3 “Some times per week”, 4 “Seldom”, and 5 “Never” and cases showing symptoms of insomnia were classified as scoring two or less on any of the items in the index. The Cronbach's alpha at 11–15 years in the profession was 0·76.

**Cognitive functions, depressive mood, and sleep quality during education**

As an indicator of cognitive functions, we used one single item from Bandura's Self-Efficacy for Self-Regulated Learning [27] “How well can you concentrate on school subjects?”. Respondents were given the options 0% “No: cannot do this at all”, 50% “Moderately can do this”, and 100% “Highly certain that I can do this”. As an indicator of symptoms of depression we used one core symptom from MDI [28] i.e. “Depressed mood”. Respondents were given the options 1 “All the time”, 2 “Most of the time”, 3 “A minor part of the time”, and 4 “Not at all”. Sleep quality was measured with one single item [29] “How do you assess your quality of sleep?”. Respondents were given the options 1 “Good”, 2 “Pretty good”, 3 “Neither good nor poor”, 4 “Pretty poor”, and 5 “Poor”.

Alt-text: Unlabelled box

## Data sharing statement

Data obtained for the study will not be accessible to others.

## Declaration of Competing Interest

All authors report no competing interests.

## Appendix Supplementary materials

Image, application 1

Image, application 2
