# Supplementary material for: Consequences of early career nurse burnout: A prospective long-term follow-up on cognitive functions, depressive symptoms, and insomnia
Source: eClinicalMedicine. 2020 Oct 5;27:100565. doi: 10.1016/j.eclinm.2020.100565 (PMC7599295; doi:10.1016/j.eclinm.2020.100565)
Supplement: Supplementary file 1 [file mmc1.docx]

1. Firth H, Britton P. "Burnout," absence and turnover amongst British nursing staff. *Journal of Occupational Psychology* 1989; **62**(1): 55-9.

2. Brown LK, Schultz JR, Forsberg AD, King G, Kocik SM, Butler RB. Predictors of retention among HIV/hemophilia health care professionals. *Gen Hosp Psychiatry* 2002; **24**(1): 48-54.

3. Hochwalder J. A longitudinal study of the relationship between empowerment and burnout among registered and assistant nurses. *Work* 2008; **30**(4): 343-52.

4. Suzuki E, Itomine I, Saito M, Katsuki T, Sato C. Factors affecting the turnover of novice nurses at university hospitals: a two year longitudinal study. *Japan Journal of Nursing Science: JJNS* 2008; **5**(1): 9-21.

5. Demerouti E, Le Blanc PM, Bakker AB, Schaufeli WB, Hox J. Present but sick: A three-wave study on job demands, presenteeism and burnout. *The Career Development International* 2009; **14**(1): 50-68.

6. Anagnostopoulos F, Niakas D. Job burnout, health-related quality of life, and sickness absence in Greek health professionals. *Eur Psychol* 2010; **15**(2): 132-41.

7. Figueiredo-Ferraz H, Grau-Alberola E, Gil-Monte PR, Garcia-Juesas JA. Burnout and job satisfaction among nursing professionals. *Psicothema* 2012; **24**(2): 271-6.

8. Rudman A, Gustavsson P, Hultell D. A prospective study of nurses' intentions to leave the profession during their first five years of practice in Sweden. *Int J Nurs Stud* 2014; **51**(4): 612-24.

9. Spence Laschinger HK, Wong C, Read E, et al. Predictors of new graduate nurses' health over the first 4 years of practice. *Nursing open* 2019; **6**(2): 245-59.

10. Hatch DJ, Freude G, Martus P, Rose U, Muller G, Potter GG. Age, burnout and physical and psychological work ability among nurses. *Occupational Medicine (Oxford)* 2018; **68**(4): 246-54.

11. Hatch DJ, Potter GG, Martus P, Rose U, Freude G. Lagged versus concurrent changes between burnout and depression symptoms and unique contributions from job demands and job resources. *J Occup Health Psychol* 2019; **24**(6): 617-28.

12. Gorgievski MJ, Van der Heijden BIJM, Bakker AB. Effort-reward imbalance and work-home interference: a two-wave study among European male nurses. *Work Stress* 2019; **33**(4): 315-33.

13. Van der Heijden B, Mahoney CB, Xu YZ. Impact of Job Demands and Resources on Nurses' Burnout and Occupational Turnover Intention Towards an Age-Moderated Mediation Model for the Nursing Profession. *Int J Env Res Public Health* 2019; **16**(11): 22.

**Supplementary Table 1.** Search strategy for each database and number of hits for each term and combinations. We searched Medline, Web of Science, PsycINFO, and CINAHL for studies on burnout, nurses, and long-term follow up that were published from the inception of each database to March 3, 2020.

| **Database** | **#** | **Search strategy term** | **Hits** |
| --- | --- | --- | --- |
| **Medline** | **Block 1: Burnout** | | |
|  | 1 | exp Burnout/ | 11569 |
|  | 2 | exp Occupational stress/ | 12665 |
|  | 3 | exp Mental fatigue/ | 1904 |
|  | 4 | (burnout* OR burn-out*).ti,ab,kf | 11586 |
|  | 5 | (burn* ADJ3 out).ti,ab,kf | 1882 |
|  | 6 | (emotional* ADJ3 exhaust*).ti,ab,kf | 2607 |
|  | 7 | (exhaustion disorder OR adjustment disorder).ti,ab,kf | 1080 |
|  | 8 | (depersonali?at* OR de-personali?at*).ti,ab,kf | 2774 |
|  | 9 | fatigue.ti,ab,kf | 92248 |
|  | 10 | Exp Stress, psychological/ | 127852 |
|  | 11 | Exp Occupational health/ | 32677 |
|  | 12 | Exp Work/ | 62149 |
|  | 13 | Workplace/ | 22104 |
|  | 14 | (Work* OR occupation* OR job OR professional*).ti,ab,kf | 1808995 |
|  | 15 | 11 OR 12 OR 13 OR 14 | 1847000 |
|  | 16 | 10 AND 15 | 30668 |
|  | 17 | (Stress ADJ3 (Work* OR occupation* OR job OR professional*)).ti,ab,kf | 12190 |
|  | 18 | 1 OR 2 OR 3 OR 4 OR 5 OR 6 OR 7 OR 8 OR 9 OR 16 OR 17 | 138555 |
|  | **Block 2: Nurses** | | |
|  | 19 | Exp Nurses/ | 86950 |
|  | 20 | Exp Students, nursing/ | 23963 |
|  | 21 | Exp Nursing staff/ | 65501 |
|  | 22 | (Nurs* OR midwi* OR mid-wi*).ti,ab,kf | 468580 |
|  | 23 | 19 OR 20 OR 21 OR 22 | 527668 |
|  | **Block 3: Long term follow-up** | | |
|  | 24 | Exp Observational study/ | 74896 |
|  | 25 | Exp Observational studies as topic/ | 1696 |
|  | 26 | Exp Cohort studies/ | 1958575 |
|  | 27 | (longitudinal OR cohort* OR follow-up OR follow up OR followup OR 5-year OR 6-year OR 7-year OR 8-year OR 9-year OR 1#-year OR 2#-year OR long-term OR long term).ti,ab,kf | 2430174 |
|  | 28 | (Repeated ADJ3 measure*) | 51387 |
|  | 29 | 24 OR 25 OR 26 OR 27 OR 28 | 3561514 |
|  | **Combination of blocks** | | |
|  | 30 | 18 AND 23 AND 29 | ***1781*** |
| **Cinahl** | **Block 1: Burnout** | | |
|  | 1 | (MH "Stress, Occupational+") | 23365 |
|  | 2 | (MH "Mental Fatigue+") | 733 |
|  | 3 | TI ( burnout OR burn-out ) OR AB ( burnout OR burn-out ) | 8690 |
|  | 4 | TI burn* n3 out OR AB burn* n3 out | 881 |
|  | 5 | TI emotional* n3 exhaust* OR AB emotional* n3 exhaust* | 1762 |
|  | 6 | TI ( "exhaustion disorder" OR "adjustment disorder" ) OR AB ( "exhaustion disorder" OR "adjustment disorder" ) | 307 |
|  | 7 | TI ( depersonali?ation OR de-personali?ation ) OR AB ( depersonali?ation OR de-personali?ation ) | 1211 |
|  | 8 | TI fatigue OR AB fatigue | 33409 |
|  | 9 | (MH "Stress, Psychological+") | 76846 |
|  | 10 | (MH "Occupational Health+") | 56290 |
|  | 11 | (MH "Work+") | 6605 |
|  | 12 | (MH "Work Environment+") | 29318 |
|  | 13 | TI ( Work* OR occupation* OR job OR professional* ) OR AB ( Work* OR occupation* OR job OR professional* ) | 594309 |
|  | 14 | 10 OR 11 OR 12 OR 13 | 631681 |
|  | 15 | 9 AND 14 | 33171 |
|  | 16 | TI ( Stress N3 (Work* OR occupation* OR job OR professional*) ) OR AB ( Stress N3 (Work* OR occupation* OR job OR professional*) ) | 7569 |
|  | 17 | 1 OR 2 OR 3 OR 4 OR 5 OR 6 OR 7 OR 8 OR 15 OR 16 | 70594 |
|  | **Block 2: Nurses** | | |
|  | 18 | (MH "Nurses+") | 205717 |
|  | 19 | (MH "Students, Nursing+") | 34230 |
|  | 20 | (MH "Nursing Staff, Hospital") | 18323 |
|  | 21 | TI ( nurs* OR midwi* OR mid-wi* ) OR AB ( nurs* OR midwi* OR mid-wi* ) | 500509 |
|  | 22 | 18 OR 19 OR 20 OR 21 | 588868 |
|  | **Block 3: Long term follow-up** | | |
|  | 23 | (MH "Prospective Studies+") | 409527 |
|  | 24 | (MH "Repeated Measures") | 49454 |
|  | 25 | (MH "Retrospective Design") | 238661 |
|  | 26 | TI ( longitudinal OR cohort* OR follow-up OR follow up OR followup OR 5-year OR 6-year OR 7-year OR 8-year OR 9-year OR 1#-year OR 2#-year OR long-term OR "long term" ) OR AB ( longitudinal OR cohort* OR follow-up OR follow up OR followup OR 5-year OR 6-year OR 7-year OR 8-year OR 9-year OR 1#-year OR 2#-year OR long-term OR "long term" ) | 582292 |
|  | 27 | TI Repeated n3 measure* OR AB Repeated n3 measure* | 20254 |
|  | 28 | 24 OR 25 OR 26 OR 27 OR 28 | 966571 |
|  | **Combination of blocks** | | |
|  | 30 | 18 AND 23 AND 29 | ***1886*** |
| Psychinfo | **Block 1: Burnout** | | |
|  | 1 | exp occupational stress/ | 21182 |
|  | 2 | fatigue/ | 8907 |
|  | 3 | (burnout* or burn-out*).ti,ab,id. | 12381 |
|  | 4 | (burn* adj3 out).ti,ab,id. | 1003 |
|  | 5 | (emotional* adj3 exhaust*).ti,ab,id. | 3628 |
|  | 6 | (exhaustion disorder or adjustment disorder).ti,ab,id. | 1181 |
|  | 7 | (depersonali?at* or de-personali?at*).ti,ab,id. | 3874 |
|  | 8 | fatigue.ti,ab,id. | 24599 |
|  | 9 | exp Stress/ | 112260 |
|  | 10 | exp occupational health/ | 3823 |
|  | 11 | exp Working Conditions/ | 28600 |
|  | 12 | exp Work Load/ | 2787 |
|  | 13 | exp "Quality of Work Life"/ | 1543 |
|  | 14 | (Work* or occupation* or job or professional*).ti,ab,id. | 946064 |
|  | 15 | 10 or 11 or 12 or 13 or 14 | 948213 |
|  | 16 | 9 and 15 | 32898 |
|  | 17 | (Stress adj3 (Work* or occupation* or job or professional*)).ti,ab,id. | 13958 |
|  | 18 | 16 or 17 | 36191 |
|  | 19 | 1 or 2 or 3 or 4 or 5 or 6 or 7 or 8 or 18 | 69542 |
|  | **Block 2: Nurses** | | |
|  | 20 | exp Nurses/ | 30754 |
|  | 21 | exp Nursing Students/ | 5045 |
|  | 22 | (Nurs* or midwi* or mid-wi*).ti,ab,id. | 18323 |
|  | 23 | 20 OR 21 OR 22 | 588868 |
|  | **Block 3: Long term follow-up** | | |
|  | 24 | Exp longitudinal studies/ | 16356 |
|  | 25 | Exp followup studies | 12371 |
|  | 26 | exp retrospective studies/ | 471 |
|  | 27 | exp cohort analysis/ | 1379 |
|  | 28 | repeated measures/ | 685 |
|  | 29 | (longitudinal or cohort* or follow-up or follow up or followup or 5-year or 6-year or 7-year or 8-year or 9-year or 1#-year or 2#-year or long-term or long term).ti,ab,id. | 420980 |
|  | 30 | (Repeated adj3 measure*).ti,ab,id. | 17498 |
|  | 31 | 23 OR 24 OR 25 OR 26 OR 27 OR 28 OR 29 | 439757 |
|  | **Combination of blocks** | | |
|  | 32 | 19 AND 23 AND 31 | ***748*** |
| Web of Science | **Block 1: Burnout** | | |
|  | 1 | burnout* or burn-out* | 28294 |
|  | 2 | (burn* near/3 out) | 4439 |
|  | 3 | emotional* near/3 exhaust* | 4396 |
|  | 4 | “exhaustion disorder” or “adjustment disorder” | 1043 |
|  | 5 | (depersonali?at* or de-personali?at* | 3542 |
|  | 6 | fatigue | 238200 |
|  | 7 | Stress near/3 (Work* or occupation* or job or professional*) | 27852 |
|  | 8 | 1 or 2 or 3 or 4 or 5 or 6 or 7 | 292480 |
|  | **Block 2: Nurses** | | |
|  | 9 | Nurs* or midwi* or mid-wi* | 18323 |
|  | **Block 3: Long term follow-up** | | |
|  | 10 | longitudinal or cohort* or follow-up or “follow up” or followup or 5-year or 6-year or 7-year or 8-year or 9-year or 1#-year or 2#-year or long-term or “long term” | 3721867 |
|  | 11 | Repeated NEAR/3 measure* | 60019 |
|  | 12 | 10 OR 11 | 3763402 |
|  | **Combination of blocks** | | |
|  | 13 | 8 AND 9 AND 12 | ***1829*** |

**Supplementary table 2.** Overview of the 13 identified longitudinal studies that investigate consequences of burnout in nurses.

| **Author, year/Country** | **Measure Burnout component** | **Consequence variable** | **Sample** | **Follow-up Time** | **Interpretation** |
| --- | --- | --- | --- | --- | --- |
| Firth et al., 1989^1^  Great Britain | MBI subscales emotional exhaustion, personal accomplishment, and depersonalisation | Sickness absence  Job turnover | 106 nurses | 2 years | Emotional exhaustion predicted sickness absence in year 1.  Depersonalization predicted turnover over 2 years. |
| Brown et al., 2002^2^  United States | MBI subscales emotional exhaustion and personal accomplishment. | Occupational retention turnover | 213 hospital nurses, physicians, mental health workers | 4 years | Burnout did not predict occupational retention. |
| Hochwalder, 2008^3^  Sweden | MBI subscales emotional exhaustion, personal accomplishment, and depersonalisation | Empowerment | 1356 registered nurses and assistant nurses | 1 year | High levels of burnout predicted low levels of empowerment in assistant nurses, but not in registered nurses. |
| Suzuki et al., 2008^4^  Japan | MBI subscales physical exhaustion, emotional exhaustion and depersonalization, and personal accomplishment. | Job turnover | 1203 full-time novice nurses | 2 years | Total MBI score and subscale physical exhaustion at baseline predicted turnover. Emotional exhaustion did not predict turnover. |
| Demerouti et al., 2009^5^  The Netherlands | MBI subscales emotional exhaustion and depersonalisation | Presenteeism | 258 staff nurses | about 1.5 years | Emotional exhaustion and presenteeism are reciprocal over time. |
| Anagnostopoulos et al., 2010^6^  Greece | MBI (low, moderate and high levels) | Short- and long-term sickness absence | 487 general hospital nurses | 1 year | Burnout (emotional exhaustion) predicted short-term sickness absence (not long-term). |
| Figueiredo-Ferraz et al., 2012^7^  Spain | MBI subscales emotional exhaustion, personal accomplishment, and depersonalisation | Work satisfaction | 316 staff nurses | 1 year | There was a negative effect of burnout (subscales emotional exhaustion and depersonalization) on work satisfaction. |
| Rudman et al., 2014^8^  Sweden | OLBI subscales exhaustion and disengagement | Occupational turnover intention | 1417 hospital nurses | 5 years | Disengagement (but not exhaustion) predicted occupational turnover intention. |
| Lashinger et al., 2018^9^  Canada | MBI subscales emotional exhaustion and cynicism | Self-rated health  Mental health  PTSD risk | 406 new graduate nurses (<3 years work experience) | 1.5 years | Cynicism was a predictor for all three health outcomes. Emotional exhaustion did not predict health outcomes. |
| Hatch et al., 2018^10^  United States | OLBI subscale exhaustion | Work ability | 402 registered nurses, licensed practical nurses, certified nursing assistants and medical assistants | 1 year | High exhaustion predicted lower work ability with age. |
| Hatch et al., 2019^11^  United States | OLBI subscale exhaustion | Depressive symptoms | 402 registered nurses, licensed practical nurses, certified nursing assistants and medical assistants | 1 year | Exhaustion predicted symptoms of depression. |
| Gorgievski et al., 2019^12^  Belgium, France, The Netherlands, Germany, Italy, Poland and Slovakia | CBI | Work-home interference | 1421 male nurses | 1 year | Exhaustion predicted increased work-home interference. |
| Van der Heijden et al., 2019^13^  The Netherlands | CBI | Occupational turnover intention | 1187 registered nurses | 1 year | Burnout symptoms predicted occupational turnover intention. |
| *Note*: MBI=Maslach Burnout Inventory; CBI=Copenhagen Burnout Inventory; OLBI=Oldenburg Burnout Inventory | | | | | |

**Supplementary Table 3.** Comparing nurses with and without a previous early career episode of burnout on long-term consequences of psychological health.

|  | **Symptom prevalence at follow-up** | | | **Associations between ECB and symptoms at follow-up** | | | | | | | |
| --- | --- | --- | --- | --- | --- | --- | --- | --- | --- | --- | --- |
|  | Cohort  n (%) | ECB  n (%) | Control  n (%) | OR 1 | 95% CI | OR 2 | 95% CI | OR 3 | 95% CI | OR 4 | 95% CI |
| 4 or more cognitive problems | 275 (11·4) | 79 (26·4) | 196 (9·3) | 3·368 | 2·493-4·550 | 2·461 | 1·764-3·434 | 2·185 | 1·519-3·142 | 2·155* | 1·365- 3·402 |
| MDI ICD diagnosis | 138 (5·7) | 38 (12·8) | 100 (4·7) | 2·804 | 1·877-4·188 | 1·727 | 1·103-2·703 | 1·038 | 0·631-1·706 | 0·836 | 0·497- 1·405 |
| KSQ Insomina index | 1020 (42·2) | 178 (59·5) | 842 (39·8) | 2·245 | 1·749-2·881 | 1·909 | 1·470-2·479 | 1·723 | 1·315-2·258 | 1·540 | 1·170- 2·028 |

*Cohort % = prevalence in the total sample; ECB % = prevalence in the subsample with a history of early career burnout (ECB); control % = prevalence in the subsample without a history of ECB. OR 1 = without correction for concurrent symptoms. OR 2 = corrected for ongoing episode of job burnout. OR 3 = corrected for ongoing episode of job burnout and the other symptoms at 11-14 years post graduation i.e. cognitive problems, depression, and sleep problems respectively. OR 4 = corrected for ongoing episode of job burnout and the other symptoms at 11-14 years post graduation i.e. cognitive problems, depression, and sleep problems respectively and indicators of previous problems (with cognitive functions, symptoms of depression, and sleep problems respectively) measured during the last semester of the nursing education program. Age, sex, and cohort were included as control variables in all logistic regression analyses, thus all odds ratios (OR1-OR4) were controlled for age, sex, and cohort. * Note that this analysis was only possible to do in two of the cohorts i.e. EX2002 and EX2004 due to that the measure was not included in the EX2006 cohort during the education program.*

**Supplementary Table 4.** Items in the cognition instrument.

| **Cognitive functions** |
| --- |
|  |
| How often do you forget something you have really made an effort to remember? |
| How often do you forget something that just happened a moment ago? |
| How often do you lose things? |
| How often have you been so absent minded that you have started multiple things at the same time? |
| How often have you had difficulties summoning the concentration needed for a certain task? |
| How often have you had difficulties to focus effectively? |
| How often have your thoughts wandered off when you have really needed to focus on a task? |
| How often have you been easily distracted? |
| How often have you had trouble following the story line in a movie? |
| How often have you had trouble figuring out how to organize your work? |
| How often have you had trouble shifting your attention from one situation to another when a situation suddenly changed? |
